# Supplementary material for: MetaRibo-Seq measures translation in microbiomes
Source: Nat Commun. 2020 Jun 29;11:3268. doi: 10.1038/s41467-020-17081-z (PMC7324362; doi:10.1038/s41467-020-17081-z)
Supplement: Supplementary file 10 — Supplementary Data 7 [file 41467_2020_17081_MOESM10_ESM.zip › File2/Confidence_High_Taxonomy/58846_out.krona.html]

Javascript must be enabled to view this page.

members
magnitude
magnitudeUnassigned
count
unassigned
taxon
rank

58846\_out

106

106
2
superkingdom

phylum
976
106

class
200643
106

171549

SRS012902\_contig\_number\_contig-100\_1072.15587SRS016541\_contig\_number\_14053SRS017701\_contig\_number\_18329SRS019381\_contig\_number\_21699SRS024492\_contig\_number\_20479SRS050026\_contig\_number\_contig-100\_18389.54728SRS064276\_contig\_number\_42795SRS075398\_contig\_number\_contig-100\_783.29451SRS1041031\_contig\_number\_31129SRS1041164\_contig\_number\_contig-100\_1152.45667SRS1054928\_contig\_number\_658SRS145308\_contig\_number\_contig-100\_884.38258SRS146764\_contig\_number\_3223SRS146813\_contig\_number\_1275SRS148319\_contig\_number\_contig-100\_974.98022
106
order
15

1853231
5
family

genus
5
283168

species

SRS019397\_contig\_number\_6116SRS022137\_contig\_number\_13726SRS077127\_contig\_number\_7006SRS893172\_contig\_number\_2575SRS893327\_contig\_number\_2917
5
28118

family
171550
3

genus
239759
3

species

SRS024549\_contig\_number\_96
1
2292190

species
1

SRS104912\_contig\_number\_4823
28117

1

SRS098571\_contig\_number\_66751
1896969
species

family
11
171552

genus
2
838

2079531
1

SRS014313\_contig\_number\_5356
species

species
1

SRS016381\_contig\_number\_69
165179

genus
1283313
1

1
76122

SRS1041090\_contig\_number\_6270
species

8
577309
genus

species

SRS013215\_contig\_number\_110SRS015264\_contig\_number\_11325SRS021948\_contig\_number\_4406SRS022071\_contig\_number\_contig-100\_1159.225763SRS050299\_contig\_number\_1235SRS058268\_contig\_number\_237SRS077086\_contig\_number\_14571SRS147766\_contig\_number\_32
8
454154

species
1

SRS143876\_contig\_number\_27459
1897039

family
2005525
17

375288
17
genus


SRS017745\_contig\_number\_3305
1
1750560
species

species
1
823

SRS147977\_contig\_number\_10754

species
1

SRS1055038\_contig\_number\_2462
2293117

species
1

SRS893172\_contig\_number\_contig-100\_1260.1261
563193

species
9

SRS015960\_contig\_number\_21842SRS019601\_contig\_number\_contig-100\_1308.170329SRS050520\_contig\_number\_contig-100\_815.22200SRS051031\_contig\_number\_12099SRS098717\_contig\_number\_19632SRS140513\_contig\_number\_9519SRS140645\_contig\_number\_265SRS142542\_contig\_number\_contig-100\_673.166315SRS893373\_contig\_number\_524
658662

species
1

SRS075878\_contig\_number\_22496
387661


SRS054905\_contig\_number\_14523SRS105082\_contig\_number\_11340SRS142923\_contig\_number\_521
3
46503
species

54
815
family

54
genus
11
816

SRS015579\_contig\_number\_23792SRS018541\_contig\_number\_contig-100\_772.92505SRS021219\_contig\_number\_contig-100\_2088.82123SRS024140\_contig\_number\_contig-100\_1401.1401SRS051882\_contig\_number\_10745SRS053335\_contig\_number\_377SRS077335\_contig\_number\_9500SRS147088\_contig\_number\_2891SRS893259\_contig\_number\_6129SRS893288\_contig\_number\_2047SRS971275\_contig\_number\_contig-100\_650.273450

4
820

SRS049446\_contig\_number\_12127SRS1041157\_contig\_number\_contig-100\_2272.48277SRS149325\_contig\_number\_9878SRS150029\_contig\_number\_contig-100\_4295.95942
species

species
2
246787

SRS052027\_contig\_number\_16067SRS1041140\_contig\_number\_207

1
821

SRS075398\_contig\_number\_765
species

species
1896974
3

SRS015663\_contig\_number\_33589SRS020394\_contig\_number\_12406SRS1041118\_contig\_number\_3872

2292921
1

SRS893382\_contig\_number\_358
species

1
469590

SRS893341\_contig\_number\_6105
species

species

SRS076976\_contig\_number\_5476SRS1041139\_contig\_number\_2811
2
28116

1
2302926

SRS1055083\_contig\_number\_contig-100\_873.82411
species

3
818

SRS015694\_contig\_number\_152SRS1041142\_contig\_number\_9080SRS104485\_contig\_number\_106
species

species

SRS1041136\_contig\_number\_22215
1
469593


SRS063040\_contig\_number\_26610SRS1041145\_contig\_number\_24162SRS893285\_contig\_number\_2709
3
28111
species

species
46506
2

SRS077454\_contig\_number\_11119SRS097889\_contig\_number\_5684


SRS055982\_contig\_number\_17613
1
2292923
species

species
47678
7

SRS024132\_contig\_number\_4756SRS075821\_contig\_number\_contig-100\_959.130815SRS1041039\_contig\_number\_contig-100\_712.151789SRS1055034\_contig\_number\_8299SRS147977\_contig\_number\_1784SRS893295\_contig\_number\_13141SRS893369\_contig\_number\_1502

species
2
457387

SRS019445\_contig\_number\_31422SRS1041143\_contig\_number\_6973


SRS013382\_contig\_number\_599
1
817
species

species
1

SRS024075\_contig\_number\_11944
310300


SRS011302\_contig\_number\_11396
1
1638779
species


SRS023829\_contig\_number\_979SRS024388\_contig\_number\_8079SRS062701\_contig\_number\_6836SRS098073\_contig\_number\_17491SRS149181\_contig\_number\_29804
5
371601
species


SRS893366\_contig\_number\_17701
1
329854
species
